# Montelukast as a novel therapeutic approach in metastatic uveal melanoma harboring a *CYSLTR2* mutation: a translational case report

**DOI:** 10.1016/j.esmoop.2025.105921

**Published:** 2025-12-06

**Authors:** D. Smarsly, N. Kreuzberg, C. Langhorst, M. Scheffler, U. Siebolts, S. Lennartz, P. Koll, A.-C. Glaser, C. Franklin

**Affiliations:** 1 Department of Dermatology and Venereology, University of Cologne, Faculty of Medicine and University Hospital Cologne, Cologne, Germany; 2 Center for Integrated Oncology (CIO) Aachen Bonn Köln Düsseldorf, University Hospital Cologne, Cologne, Germany; 3 Clinic for Internal Medicine I, Hemato-Oncology, University of Cologne, Faculty of Medicine and University Hospital Cologne, Cologne, Germany; 4 Department of Pathology, University of Cologne, Faculty of Medicine and University Hospital Cologne, Cologne, Germany; 5 Department of Radiology, University of Cologne, Faculty of Medicine and University Hospital Cologne, Cologne, Germany

**Keywords:** uveal melanoma, *CYSLTR2* mutation, montelukast, leukotriene receptor, targeted therapy, case report, drug repurposing

## Abstract

**Background:**

Uveal melanoma (UM), the most common primary intraocular malignancy in adults, has limited systemic treatment options in the metastatic setting. Recent insights into cysteinyl leukotriene receptors (CysLTRs)—particularly *CYSLTR2* mutations (prevalence 2%-4%)—suggest new therapeutic approaches for patients who progress despite standard therapies.

**Case:**

We report the case of a 59-year-old male with metastatic UM harboring a *CYSLTR2* mutation. The patient experienced progression after multiple systemic treatments, including immune checkpoint inhibitors (ipilimumab/nivolumab, pembrolizumab), chemotherapy (dacarbazine, gemcitabine/treosulfan), and local radiotherapy. Lacking human leukocyte antigen-A∗02:01, he was ineligible for tebentafusp. In November 2022, next-generation sequencing identified a *CYSLTR2* mutation. Based on molecular tumor board recommendation, off-label treatment with montelukast, a selective CysLT1 receptor antagonist, was initiated in March 2024. At that time, the patient had widespread metastases. Montelukast led to sustained disease stabilization for >12 months, with excellent tolerability and no reported adverse events. The observed effect may be explained by inhibition of CYSLTR1 and modulation of CYSLTR2 signaling in the mutated receptor context.

**Conclusion:**

This is the first published case suggesting a potential role for leukotriene receptor antagonists in *CYSLTR2*-mutant UM. These findings support further preclinical and clinical investigation of montelukast as a repurposed therapy in this challenging disease entity.

## Highlights

• First clinical report of montelukast use in metastatic UM with a *CYSLTR2* mutation.
• Off-label montelukast achieved >12 months of disease stabilization in a heavily pretreated patient.
• The patient experienced no adverse events; therapy was well tolerated and orally administered.
• *CYSLTR2* mutation drives Gαq signaling and may render tumors sensitive to leukotriene pathway inhibition.
• Montelukast is a cost-effective, repurposed drug with potential in molecularly defined UM subgroups.

## Introduction

Uveal melanoma (UM) is the most common primary intraocular malignancy in adults, accounting for ∼3.8% of all melanomas. The prognosis is poor, primarily due to the high risk of liver metastases and the limited systemic treatment options available.^1^

The bispecific antibody tebentafusp is the only therapeutic agent to date that has demonstrated an improvement in overall survival in a prospective randomized study in patients with metastatic UM.^2^ Its use is restricted to patients who are human leukocyte antigen (HLA)-A∗02:01-positive, however. For patients lacking this HLA type, immune checkpoint inhibitors like programmed cell death protein-1-directed antibodies in combination with cytotoxic T lymphocyte antigen-4 inhibitors can be effective in selected cases.^3^ Additional standard treatment options include liver-directed strategies, like isolated hepatic perfusion, hepatic artery infusion, transarterial chemoembolization, selective internal radiotherapy, and immunoembolization. These options for patients with liver-limited metastases, typically offer only temporary disease control.^4^ For patients experiencing disease progression despite these limited therapeutic options, there is an urgent need for new and effective treatment strategies.

Molecular profiling of UM has revealed distinct genetic alterations, including mutations in *GNAQ*, *GNA11*, *BAP1*, *CYSLTR2*, and *PLCβ4*.^1^ Cysteinyl leukotriene receptors (CysLTRs), originally implicated in inflammatory diseases, have gained increasing attention in oncology.

The cysteinyl leukotriene receptor 1 (CysLT1-R) is overexpressed in various tumor types, and its pharmacological blockade has shown antitumor effects in several preclinical models. In UM, high expression of CysLT1-R correlates with reduced melanoma-specific and overall survival.^5^^,^^6^

*CYSLTR2* mutations—found in ∼2%-4% of UM cases—represent an early oncogenic event, particularly in tumors that are wild-type for *GNAQ* and *GNA11*. These mutations lead to constitutive activation of the receptor and downstream endogenous Gαq signaling, thereby promoting tumorigenesis *in vivo*. *CYSLTR2* mutations therefore converge on the same oncogenic signaling pathways that are activated by *GNAQ* or *GNA11* mutations.^7^, ^8^, ^9^

Montelukast, an FDA-approved CysLT1-R antagonist with an excellent safety record in the treatment of bronchial asthma for >20 years, has demonstrated dose-dependent antiproliferative, pro-apoptotic, and immunomodulatory *effects in vitro* and *in vivo* across various cancer models. It has not yet been evaluated in the context of UM, however. Notably, murine studies have shown that montelukast may also modulate the expression of cysteinyl leukotriene receptor 2 (CysLT2-R), suggesting potential therapeutic relevance in *CYSLTR2*-mutant cancers.^6^^,^^10^

Here, we present the first clinical evidence supporting a durable response to montelukast in a patient with metastatic UM harboring a *CYSLTR2* mutation.

## Case presentation

A 59-year-old male was diagnosed with UM in 2010 after presenting with visual disturbances. Enucleation of the right eye was carried out, and no metastases were detected at the time of initial staging.

In July 2021, the patient presented with left flank pain. Imaging revealed a large abdominal mass, which was histologically confirmed as a metastasis of UM. After surgical resection, first-line systemic therapy with ipilimumab and nivolumab was initiated. Treatment had to be discontinued after four cycles, however, due to severe immune-related hepatitis (Common Terminology Criteria for Adverse Events grade III-IV).

**Figure 1:**
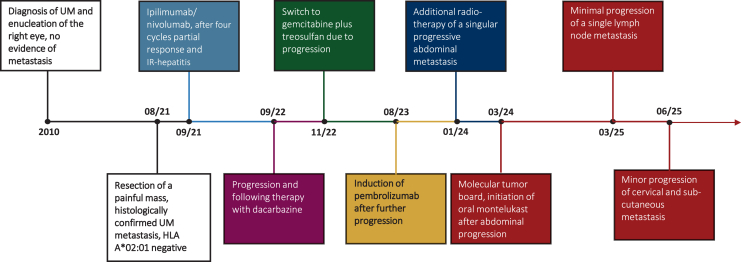
**Therapy timeline.** The timeline depicts the sequence and duration of therapeutic interventions administered for treatment of the metastatic uveal melanoma.

Partial remission was initially achieved, but in August 2022 disease progression was observed, including the development of cerebral metastases. Due to a negative test for HLA-A∗02:01, the patient subsequently received dacarbazine, followed by gemcitabine and treosulfan due to further progression. Subsequent immunotherapy with pembrolizumab resulted in a mixed response. Additionally, local radiotherapy (30 gray) was administered to an enlarging abdominal metastasis (Figure 1).

In November 2022, next-generation sequencing revealed a *CYSLTR2* mutation (Supplementary Table S1, available at https://doi.org/10.1016/j.esmoop.2025.105921). The case was reviewed by the institutional molecular tumor board, which recommended off-label therapy with montelukast (10 mg orally, once daily). Treatment was initiated in March 2024. At that time, computed tomography imaging revealed pulmonary, peritoneal, renal, and lymph node metastases. LDH (281 U/l; R <250 U/l) and S100 (0.62 μg/l; R <0.15 μg/l) concentrations were slightly elevated, while liver enzymes remained within the normal range. Despite the advanced stage of disease, the patient was in good general condition, with an Eastern Co-operative Oncology Group performance status of 0.

**Figure 2:**
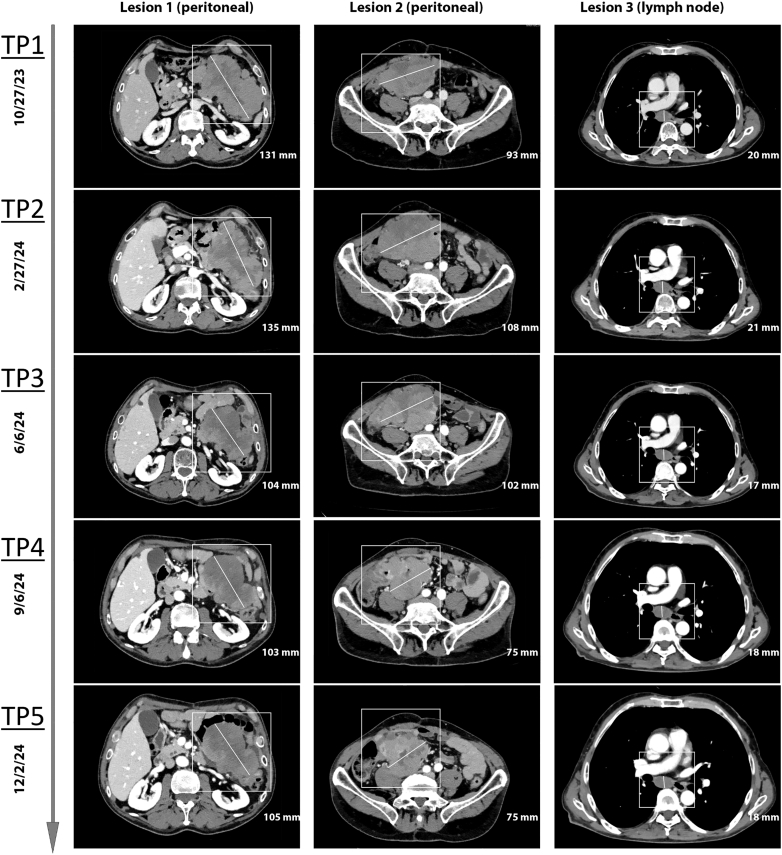
**Longitudinal imaging of three metastatic lesions in a patient with uveal melanoma harboring a CYSLTR2 mutation, refractory to standard therapies, receiving montelukast.** Timepoint (TP) 1 represents the second-latest follow-up before therapy initiation with montelukast, TP2 the baseline immediately before montelukast, and TP3-TP5 represent follow-up examinations during treatment. The first column depicts a large peritoneal metastasis in the right lower abdominal quadrant, which increased in size between TP1 and TP2, then decreased after treatment initiation and remained stable thereafter. The second column shows a large peritoneal metastasis in the left upper abdominal quadrant with initial growth from TP1 to TP2, mild regression at TP3, pronounced shrinkage at TP4, and subsequent stability at TP5. The third column illustrates a subcarinal lymph node metastasis demonstrating subtle regression at TP3 followed by stable size through the remaining follow-ups.

Since the initiation of montelukast, imaging carried out at 3-month intervals has demonstrated stable disease. The therapy has been well tolerated, with no adverse events reported to date (Figure 2). As of March 2025, only minor progression of a single supraclavicular lymph node metastasis was observed. Follow-up staging in June 2025 revealed minimal additional progression involving cervical and subcutaneous metastases. The patient remains under close clinical surveillance.

## Informed consent

Written permission for the publication of the case report has been granted.

## Discussion

Systemic treatment options for metastatic UM remain limited and cost-intensive, particularly for patients who are HLA-A∗02:01-negative and thus ineligible for tebentafusp. Dual immune checkpoint blockade with ipilimumab and nivolumab yields modest objective response rates (11.6%-18%) and short progression-free survival (2.7-5.5 months), with high rates of immune-related adverse events.^11^, ^12^, ^13^ Conventional chemotherapies, such as dacarbazine or gemcitabine, in combination with treosulfan offer minimal and transient benefit, highlighting the urgent need for more effective and better-tolerated therapies in this setting.^14^^,^^15^ Altogether, current standard-of-care therapies are frequently associated with considerable toxicity and require intensive monitoring, which can significantly affect patients’ quality of life. Both systemic and liver-directed treatment approaches—such as tebentafusp, immune checkpoint inhibitors, or isolated hepatic perfusion—also entail substantial financial costs, limited availability, and the need for specialized infrastructure. These clinical and logistical barriers restrict access for many patients, particularly outside tertiary care centers or in resource-limited regions, further emphasizing the importance of affordable, well-tolerated, and widely accessible therapeutic alternatives.

In the presented case, montelukast achieved sustained disease stabilization over at least 14 months in a patient with heavily pretreated *CYSLTR2*-mutant UM. This outcome notably exceeded the clinical benefit seen with previous therapies in both duration and tolerability. Preclinical data suggest that montelukast may suppress tumor growth through two potential mechanisms: firstly, by antagonizing CysLT1-R signaling pathways involved in tumor proliferation, survival, and inflammatory signaling—independent of mutational background^6^; and secondly, by modulating or indirectly antagonizing CysLT2-R expression, which may be particularly relevant in *CYSLTR2*-mutant tumors.^10^

From an immunological perspective, montelukast may also modulate the tumor microenvironment beyond its direct effects on tumor cells. Leukotriene signaling via CysLT1-R has been associated with the recruitment of immunosuppressive myeloid cells, increased Th2 cytokine production, and regulatory T-cell infiltration—mechanisms that collectively contribute to immune evasion and reduced cytotoxic T-cell function. Inhibition of this pathway may help restore antitumor immune activity.^16^ Although the immunomodulatory effects of CysLT1-R are better characterized, evidence from allergic and tumor models suggests a regulatory role for CysLT2-R. CysLT2-R deficiency enhances Th2-type immune responses *in vivo*, implying an immunosuppressive capacity of the receptor.^17^^,^^18^ Furthermore, lower CysLT2-R expression has been linked to more aggressive tumor behavior in patient tumor arrays, supporting a potential protective role against progression.^19^ While the exact molecular mechanisms remain to be elucidated, this dual activity provides a compelling rationale for repositioning montelukast as a low-toxicity therapeutic approach in selected UM patients. Notably, the drug’s oral availability, established safety profile, and widespread clinical use further support its feasibility for clinical repurposing.^20^ In addition, unlike conventional oncologic therapies, montelukast is a widely available and cost-effective medication, further supporting its potential role—particularly in resource-limited settings. We encourage routine genetic testing for *CYSLTR2* mutations in patients with metastatic UM who show progression after standard-of-care therapies, given the potential clinical relevance of these mutations in metastatic UM.

Limitations of this report include the off-label use of montelukast, lack of pharmacodynamic biomarker data, and the inherent constraints of a single-patient observation. As such, causality between treatment and disease control cannot be conclusively established. The temporal association, preclinical plausibility, and clinical trajectory collectively support further investigation nevertheless.

## Conclusion

This case suggests that montelukast—a well-tolerated, orally available CysLT1-R antagonist—may offer clinical benefit in patients with metastatic UM, particularly those with *CYSLTR2* mutations. Given the lack of effective treatment options in this population, cysteinyl leukotriene receptor antagonists warrant further preclinical and clinical exploration as a targeted therapeutic strategy in UM.
