# Supplementary material for: Montelukast as a novel therapeutic approach in metastatic uveal melanoma harboring a CYSLTR2 mutation: a translational case report
Source: ESMO Open. 2025 Dec 6;11(1):105921. doi: 10.1016/j.esmoop.2025.105921 (PMC12741292; doi:10.1016/j.esmoop.2025.105921)
Supplement: Supplementary data [file mmc1.docx]

**Supplementary Data**

**Table 1: 11/2022 Mutation analysis using parallel sequencing (Next Generation Sequencing, NGS)**

| **Gene** | **NM Number** | **Exon** | **Mutation Status** | **Freq. %** | **Coverage** | **Tumour Interpretation** |
| --- | --- | --- | --- | --- | --- | --- |
| **BRAF** | **NM_004333** | **11, 15** | **Wild type** |  |  |  |
| **CYSLTR2** | **NM_002067** | **1** | **EX5: c.386T>A p.L129Q** | **69.2** | **693** | **activating (http://ckb.jax.org)** |
| **GNA11** | **NM_002067** | **5** | **Wild type** |  |  |  |
| **GNAQ** | **NM_002072** | **4, 5** | **Wild type** |  |  |  |
| **KIT** | **NM_000222** | **8–11, 13–15, 17, 18** | **Wild type** |  |  |  |
| **NF1** | **NM_002524** | **1-58** | **EX32: c.4307_4311delinsAG p.E1437del** | **92.4** | **931** | **Deletion, likely loss of function (http://oncokb.org)** |
| **NRAS** | **NM_002524** | **2-4** | **Wild type** |  |  |  |
| **PDGFRA** | **NM_006206** | **12–15, 18** | **Wild type** |  |  |  |
| **RAC1** | **NM_018890** | **2** | **Wild type** |  |  |  |

Following enzymatic fragmentation of the DNA, the preparation and enrichment of the fragment library were performed using a hybrid-capture method with reagents from Twist Bioscience. To enrich the gene regions, custom-designed oligonucleotide probes targeting a melanoma panel were used. Sequencing was carried out using Illumina sequencing platforms.

The subsequent data analysis was conducted using the following thresholds:

• Allele frequency (AF): 5%

• Read depth/coverage (COV): 200x

Result for Block I:

Tumor cell content: 90%
